# Online Andrology: Real‐World Status Quo of Patients’ Online Information Sources and Comparison With Large Language Models in Germany

**DOI:** 10.1111/andr.70286

**Published:** 2026-06-22

**Authors:** Traeger Max, Gloeckler Paulina, Hess Jochen, Kranz Jennifer, Morgenstern Saskia, Himmelsbach Ruth, Gratzke Christian, Schlager Daniel

**Affiliations:** ^1^ Clinic of Urology University Hospital Freiburg Freiburg Germany; ^2^ Clinic of Urology Pediatric Urology and Uro‐Oncology University Hospital Essen Essen Germany; ^3^ Department of Urology and Pediatric Urology University Hospital RWTH Aachen Aachen Germany; ^4^ Department of Urology and Kidney Transplantation Martin Luther University Halle (Saale) Germany; ^5^ Clinic of Urology Agaplesion Markus Hospital Frankfurt am Main Germany

**Keywords:** andrology, digital health, erectile dysfunction, online information, patient information, readability

## Abstract

**Background:**

The internet, including websites and large language models (LLMs), is an increasingly important information resource for medical patients. However, information quality varies, potentially leading to misinformation. Andrological patients may particularly rely on online sources due to the sensitive nature of their conditions.

**Objectives:**

To assess the prevalence and quality of internet research among andrological patients and evaluate common online sources for comprehensibility, readability, and accuracy.

**Materials and Methods:**

Patients (*n* = 283) at four German andrological centers completed a questionnaire on their online information behavior between November 2022 and October 2023. Common online sources were objectively evaluated using the DISCERN tool and Flesch readability index in 2023. Popular LLMs were also assessed in October 2024 and compared to traditional websites.

**Results:**

67% (*n* = 190/283) of andrological patients seek medical information before appointments, primarily using the internet (52%, *n* = 148/283) and general practitioners (49%, *n* = 139/283). Patients under 50 predominantly use online sources (60%, *n* = 74/122). Official medical association websites and Wikipedia are preferred, but 30% also use commercial sites (*n* = 91/283). In general, most common websites and LLMs provide sufficient information but lack easy comprehensibility and readability.

**Discussion:**

The study highlights the widespread use of online resources by andrological patients and emphasizes the importance of high‐quality medical information. While online tools show promise, the value of physician–patient communication remains irreplaceable and cannot be replaced by chatting with LLMs.

**Conclusion:**

Official medical association should provide accurate and easily understood information for andrological patients. Efforts to improve the online media literacy and communication skills of medical personnel are necessary.

**Trial Registration Number:**

The study was registered in the German WHO primary registry, the German Clinical Trials Register (DRKS) (Number: DRKS00029651).

## 1 Introduction

The internet has become a primary source of medical information for patients, significantly impacting patient‐provider interactions. This trend influences the interaction between healthcare personnel and patients, offers opportunities for enhanced shared decision‐making due to better understanding of pathophysiology, treatment and prevention, and therefore may also influence patients’ offline medical behaviors [1, 2, 3, 4]. As an example for western countries, in 2018 nine out of ten people in the United States used the internet and 75% of them were searching for medical information [5]. Nevertheless, online sources might pursue diverse objectives (e.g., commercial interests) and may not consistently meet the necessary standards of evidence‐based medicine and unbiased information [6, 7]. Besides context quality issues, medical information focusing on patients must be comprehensible and readable [8]. The National Institutes of Health, the American Medical Association, and the US Department of Health and Human Services state that online medial information for patients should be readable for a pupil in sixth grade or even easier [9]. Therefore, it is crucial for healthcare providers to know their patients’ sources of information to assist in identifying misinformation and promoting a better comprehension of their concerns.

Recently, large language models (LLMs) have emerged as a new resource for patients seeking medical information [10, 11]. In comparison to traditional websites, the texts are not written by human experts but are generated live by artificial intelligence (AI). While offering personalized responses, LLMs raise similar concerns about quality, comprehensibility, and accuracy as traditional websites.

Andrology presents a prominent case in online health information seeking. Men, who generally utilize healthcare services less frequently than women [12], often face sensitive issues in andrology that may lead to further hesitation in seeking in‐person care due to apprehension about potential social stigma. Consequently, andrology is a primary focus for patients researching urological conditions online via traditional websites and LLMs [13, 14, 15].

This study employed an anonymous survey across four German andrological centers to investigate online health information seeking behaviors among andrological patients. The research aimed to identify common online resources used by these patients. The study evaluated the readability, comprehensibility, and medical accuracy of these sources using validated tools (DISCERN, Flesch‐index) and expert assessment. Additionally, patients provided subjective ratings of their utilized online sources. Eventually, popular LLMs were consulted and their answers were also analyzed and compared to traditional websites.

## 2 Material and Methods

### 2.1 Study Collective

Patients were recruited from andrological consultations at specialized sections of University Hospitals in Aachen, Essen, and Freiburg, as well as Agaplesion Markus Hospital Frankfurt, between November 2022 and October 2023. German‐speaking patients aged 18 and older were invited to participate. Participation was voluntary and uncompensated.

### 2.2 Study Design

The study was conducted as a prospective multi‐center cross‐sectional survey study. Primary endpoints: usage and subjective rating of online sources by patients. Secondary endpoints: objective evaluation of the most common sources and comparison with LLMs.

### 2.3 Methods

#### 2.3.1 Questionnaire

The German‐language questionnaire (see Supporting information S1) included questions about age, educational background, and reason for the medical visit, followed by inquiries about the information‐gathering habits. Questions were structured as binary or multiple‐choice, with free‐text options for selected items. Participants could select multiple answers where applicable.

Pre‐selected websites in the survey were identified through internal online research and included the most accessed sites from search engine results, covering specialized information, general information, and commercial services. Options included websites of the German Society of Urology (DGU), European Association of Urology (EAU), German health insurance providers, Wikipedia, digital publications like Men's Health and Apotheken Umschau (a German pharmacy magazine), and commercial German health platforms such as Netdoktor.de and KRY.de. Eventually, participants could rate the quality of information, clarity, and visual presentation of the websites using numerical scales from 1 to 10.

The survey questions were independent, allowing for evaluation even if the questionnaire remained partially incomplete.

#### 2.3.2 Objective Quality Assessment Using DISCERN

The DISCERN tool is a standardized instrument for evaluating the quality of written patient information on medical topics. It consists of 16 questions focusing on the efficacy of information transmission and on reliability and quality of information presentation, rather than to authenticate the accuracy of the information content [16]. For this study, the German version of DISCERN was utilized [17]. Each of the first 15 questions is rated on a 5‐point scale: 1 indicates “no,” 2–4 “partially,” and 5 “yes,” based on the presence of the specific quality criterion. Higher scores reflect better information quality. The 16th question assesses overall quality, with 1 point indicating “low—significant shortcomings,” 2–4 points “moderate—potentially important but not significant shortcomings,” and 5 points “high—minimal shortcomings”. The total DISCERN score ranges from 16 to 80 points, with higher scores indicating better quality.

#### 2.3.3 Flesch‐Index

The Flesch‐index (German version) is used to evaluate the text readability by assigning a numerical value to represent clarity and comprehensibility. It is essential to emphasize that the evaluation solely focuses on the structural aspects of the text and does not consider the content or significance. The formula typically yields a score between 0 and 100, with higher scores indicating easier readability (see Table 1). Occasionally, scores may fall outside this range. The German Flesch formula is: Flesch value = 180 − ASL − (58.5 * ASW). ASL is the average sentence length and ASW is the average number of syllables per word. These metrics are computed with the aid of a language‐specific word factor [18].

**TABLE 1:** Overview of Flesch value assessments for readability.

Flesch score	Rating of the readability	School level (US)
0–30	Very difficult	College graduate
30–50	Difficult	College
50–60	Moderately difficult	10–12th grade
60–70	Average	8–9th grade
70–80	Moderately easy	7th grade
80–90	Easy	6th grade
90–100	Very easy	5th grade (11 years of age)

#### 2.3.4 Assessment of Medical Accuracy

A 5‐point Likert scale was used to assess the correctness and completeness of information provided by top sources for each clinical scenario. The scale ranged from 1 (not at all) to 5 (completely). Two independent urology specialists conducted the evaluation, assessing adherence to medical standards in accordance with German (Association of Scientific Medical Societies, AWMF) and European (European Association of Urology) guidelines.

#### 2.3.5 Evaluation of Large Language Models

The four most popular LLMs (ChatGPT, Perplexity AI, Microsoft Copilot, and Google Gemini) were evaluated using the same methodology as applied to the traditional websites. For each clinical scenario, three questions addressing diagnostics, therapy, and prognosis were formulated as plain language queries (see Supporting information S2).

### 2.4 Statistical Analysis

Descriptive statistical analysis was conducted using Microsoft Excel. Various statistical tools such as counting function, conditional counting function, percentage calculation, mean calculation, and standard deviation were applied for data evaluation. Statistical significance was determined through *p*‐value analysis using the Chi‐square test and the unpaired *t*‐test.

### 2.5 Ethics and Data Safety

Ethical approval was given by the ethics committee of Albert‐Ludwigs‐University Freiburg (application number: 22‐1337‐S1). The study was registered in the German WHO primary registry, the German Clinical Trials Register (DRKS) (Number: DRKS00029651).

The answers on the questionnaires were completely anonymous without the collection of identifiable personal data. Involvement in the research was completely voluntarily and entailed no advantages or hazards for the research subjects.

## 3 Results

### 3.1 Study Collective

Two hundred eighty‐three individuals (mean age 49.6 ±16.5 years) were included in the study. The most prevalent andrological issues reported were erectile dysfunction (42.8%), Induratio penis plastica/penile deviation (23.0%), and fertility concerns (8.5%) as outlined in Table 2. Significant age differences were observed across conditions, indicating that patients with erectile dysfunction and/or penile deviation were 17.5 years older than those with fertility issues, varicocele, or premature ejaculation (*p* < 0.0001). Educational backgrounds ranged from no formal education to doctoral degrees (Supporting information S3).

**TABLE 2:** Demographics of the study collective.

	Age in years [Average±SD]
**Andrological complaint**	
Total *n = 283 (100%)*	49.6 ± 16.5
Erectile Dysfunction *n = 121 (42.8%)*	53.4 ± 14.9
Induratio penis plastica/penile deviation *n = 65 (23.0%)*	53.9 ± 12.1
Fertility problems *n = 24 (8.5%)*	38.7 ± 10.8
Testicular bulge *n = 21 (7.4%)*	50.6 ± 16.9
Testosteron deficiency/hypogonadism *n = 16 (5.7%)*	48.9 ± 14.9
Ejaculation praecox/premature ejaculation *n = 14 (4.9%)*	37.7 ± 14.8
Penile and/or scrotal skin defects or wounds *n = 11 (3.9%)*	47.2 ± 23.2
Varicocele *n = 11 (3.9%)*	28.6 ± 12.7
Other *n = 48 (17.0%)*	45.4 ± 18.0
Not specified *n = 7 (2.5%)*	63.6 ± 7.3

### 3.2 Patients’ Online Research

**FIGURE 1:**
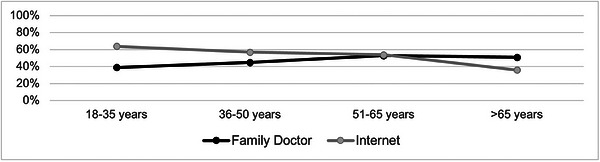
Age‐dependent preference of information source.

The majority of participants (67%) acquired information about their medical issues before their appointment. Age‐related disparity was observed: 75% of participants under 35 sought prior information compared to 51% of those over 65 (*p* = 0.0266). Primary sources of information were general practitioner (49%) and the internet (52%), with again age‐specific variations: patients under 50 primarily used the internet (60.7%), while those over 50 mostly consulted their general practitioner (*p* = 0.0329) (Figure 1). A similar pattern is also evident in the utilization of search engines: 64% of patients under 35 used them, compared to only 36% of patients over 65 (*p* = 0.0012). Especially patients suffering from sexual dysfunctions (erectile dysfunction, induration penis plastica, premature ejaculation) seek internet‐based information compared to patient with other andrologic complaints (57.5% vs. 43.4%, *p* = 0.0301). 23% of participants also utilized video‐based online material for their research.

Analysis of common internet sources revealed that respondents primarily accessed professional domains, with the German Society of Urology being the most popular at a rate of 29.5%, succeeded by the public encyclopaedia Wikipedia at 20.2%. Commercial providers collectively accounted for 29.5% of usage, while the European Association of Urology website was used by 4.9% of participants. However, the majority of respondents did not disclose specific information sources, limiting this assessment's validity (Supporting information S4).

### 3.3 Patients’ Rating of Online Sources

Only 15.9% of participants provided a valid assessment online sources. The German Society of Urology website (*n* = 18) received ratings of 7.3/10 for information and clarity, and 6.9/10 for visual presentation. Wikipedia (*n* = 15) scored slightly higher with 8.1/10 for information and clarity, and 7.8/10 for visual presentation.

### 3.4 Objective Rating of Homepages and Large Language Models

#### 3.4.1 DISCERN

**FIGURE 2:**
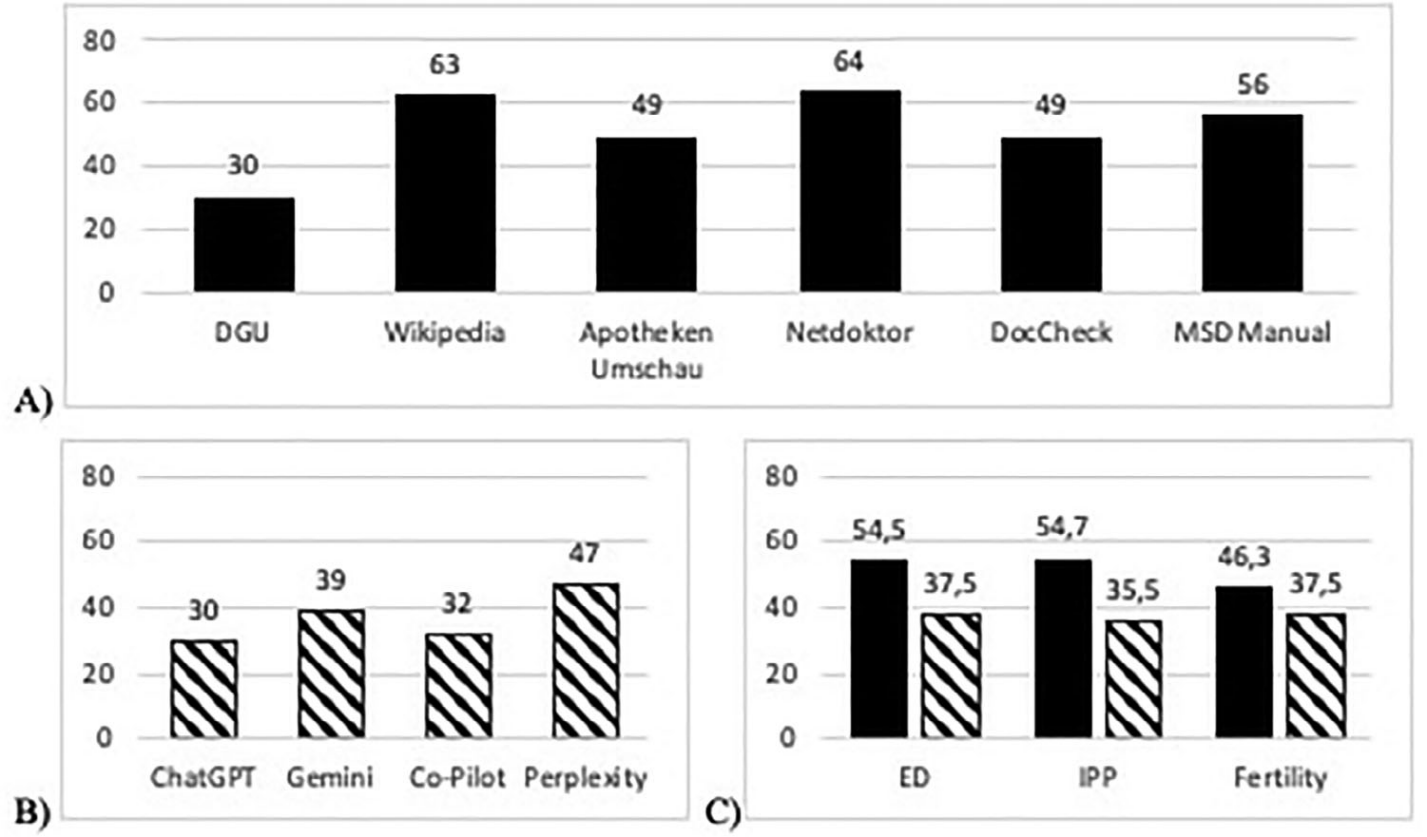
Results of the DISCERN analysis. (A) Average of websites, (B) average of LLMs, (C) comparison between websites and LLMs. LLMs, large language models.

Popular websites pertaining to the three most prevalent andrological issues (erectile dysfunction, induration penis plastic, and fertility problem) were evaluated utilizing the DISCERN tool (Supporting information S5–S7). Commercial entities and Wikipedia generally outperformed the German Society of Urology across all conditions (Figure 2). Website scores ranged from 22 (German Society of Urology on IPP) to 71 (Wikipedia and DocCheck on IPP) out of 80 points. Main reasons for low scores included missing disclosure of sources, time, or author information. LLMs scored lower ranging from 27 (ChatGPT on IPP) to 52 (Perplexity on IPP). Websites showed significantly higher overall scores compared to LLMs (51.8 vs. 36.8, *p* = 0.0018).

#### 3.4.2 Flesch

**FIGURE 3:**
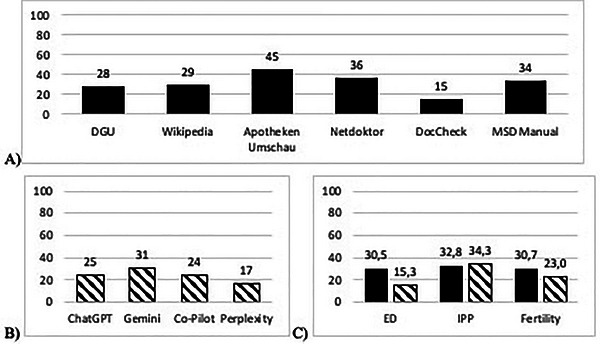
Results of the Flesch analysis. (A) Average of websites, (B) average of LLMs, (C) Comparison between websites and LLMs. LLMs, large language models.

Readability assessment using the Flesch index yielded similar outcomes (Figure 3, Supporting information S8–10). Only one homepage (Apotheken Umschau on fertility) achieved a Flesch score >50, indicating moderately difficult readability understandable for a pupil in tenth to twelfth grade. A Flesch score >80 indicates readability for pupil in sixth grade, described as conversational English for consumers.

All other resources, including patient‐focused sections of the German Society of Urology website and commercial entities, were challenging to read. LLMs scored even lower with a maximum of 41 (Gemini on IPP). Overall scores showed no significant difference between websites and LLMs (31.3 vs. 24.2, *p* = 0.0970).

#### 3.4.3 Medical Accuracy

**FIGURE 4:**
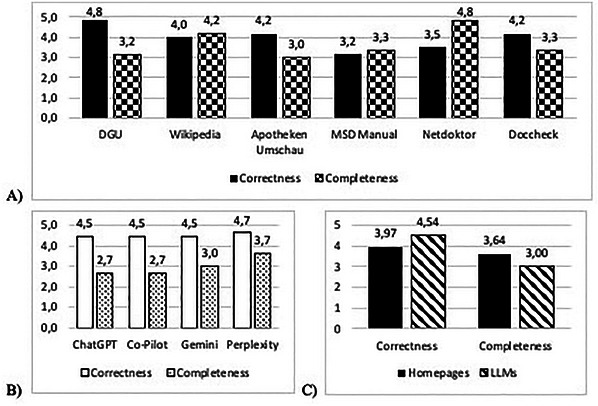
Results of the medical accuracy. (A) Average of websites, (B) average of LLMs, (C) comparison between websites and LLMs. LLMs, large language models.

Two independent urologists showed high consistency in their analysis, with 83.3% (50/60) of ratings differing by one point or less (Figure 4). Likert scale analysis from 1 to 5 showed comparable performance in correctness and completeness of medical information across all websites and LLMs. The German Society of Urology website scored highest in correctness (4.8), while Perplexity led LLMs in completeness (3.7). Overall, websites outperformed LLMs in completeness (3.64 vs. 3.00, *p* = 0.0330), but LLMs scored higher in medical correctness (4.54 vs. 3.97, *p* = 0.0233).

## 4 Discussion

This study validates that individuals with andrological complaints frequently initiate their medical information search online, particularly among younger demographics, potentially because they are used to online research and see the internet as the “natural first information source” [19]. In this study, 64% of patients under 35 used the internet as their primary information source prior to the medical appointment. Despite the Internet's dominant position, the quality of online information tends to be mediocre and capricious. This finding is in line with current research in other medical fields, such as recent studies on the quality of online information on inferior vena cava filters [20], on pain [9], or on spinal cord stimulation [5]. It appears that substandard quality is not limited to commercial websites but extends to the official websites of professional organizations, which often fall short of the necessary standards regarding readability and comprehensibility despite targeting the general public and patients. The patient information on the website of the German Society of Urology achieved only the lowest scores in comprehensibility.

Starting an online search for medical information, many individuals typically employ a search engine (49% of participants in this study). However, identifying websites that offer pertinent information among the search results can be daunting, especially when some sites appear at the top due to advertising agreements with search engine operators that cannot easily identified by more than half of users [21]. Research shows that 99% of the clicks go for the top ten results with more than half of them for the top search result. Quality seals like the “Health on the Net Foundation (HON)” have attempted to denote trustworthy websites, but issues with counterfeiting and inconsistent quality have led to their decline [22]. Currently, there is a lack of a reliable quality seal for medical information on the internet. Furthermore, the content on certified websites often fails to exhibit superior quality compared to unverified sources [23]. This study found overall sufficient but still improvable medical accuracy (3.97/5) and completeness (3.64/5) for the medical information on analyzed websites.

The emergence of chatbots utilizing AI is altering the landscape of information retrieval once again by providing targeted information and personalized responses. Various investigations have demonstrated the promising outcomes of information dissemination through chatbots. Nonetheless, the issue of readability and comprehensibility remains a concern. Consequently, platforms like “Chat‐GPT” are only partially suitable as tools for patient information as showed by Gordon et al. on questions of medical imaging [24] or by Ozduran et al. on pain [25] or by Kara et al. on Ankylosing spondylitis [26]. Our study showed that the comprehensibility and readability of LLM answers are not satisfactory with overall DISCERN score of 36.8/80 and Flesch‐Score of 24.2/100. A thorough analysis of AI answers on erectile dysfunction by Şahin et al. showed similar results [15]. Notably, exact prompt formulation is crucial for LLM responses [27], making them particularly difficult to evaluate in research projects, because the answers are highly individual. Further problems of current LLMs are the underrepresentation of minorities in the datasets employed to formulate AI algorithms and the delay in using the most current information [28]. Moreover, the transparency of primary sources is not always readily apparent, yet these sources are vital for validating the information utilized by AI systems. A solution for this can be the development of specialized chatbots like the system “UroBot” that only uses European guidelines as a primary source and shows significant better information than ChatGPT [29].

An alternative method for offering easily comprehensible information online is through medical topic videos that were used by 23% of participants in this study. Videos offer the advantage of visual aids, enhancing comprehension, but are not without drawbacks: Navigating longer videos without timestamps can impede swift and targeted information retrieval. This process may be more time‐intensive than scanning text and may result in a higher cognitive load when watching videos [30]. Furthermore, identifying primary sources within video content is not always straightforward. Their medical accuracy is not always guaranteed as Toprak et al. showed for online videos on the topic of delayed ejaculation [31]. Hanci et al. showed that only 3% of the online videos on percutaneous tracheostomy contained completely sufficient data [32].

Despite all those promising developments for the individual medical information of patients, the medical doctors and the physician–patient relationship remain of highest importance. This study showed that nearly 50% of all participants relied in their general practitioner as the main source of information. Even 39% of patients under 35 trust their doctor as a first source of information. Moreover, all of the participants were patients in an andrological consultation stating that they are looking for more information and guidance and do not rely only on their internet sources. Bad communication of physicians leads to worse therapy adherence [33], while good communication leads not only to better patient information but also to patient empowerment playing a pivotal role in shared decision making and to better health outcomes [34, 35]. A combination of well‐informed patients via online sources together with stable physician–patient relationship can lead to optimal decisions and clinical outcome [36]. Keeping this in mind, there is a desperate need for good education on communication skills and online media literacy for medical students, nurses and physicians [37, 38].

## 5 Strengths and Limitations of This Study

This study gives a broad view on online patient information in German andrology including a remarkable number of patients. Until now most publications on this topic focus on English speaking patients and information. Furthermore, it combines the strengths of a subjective patient survey and objective evaluation of online sources. Due to the focus on German the results may not be representative for websites in other languages. Voluntary participation and the “referral consultation” setting may have favored well‐informed patients, potentially facilitating a selection bias. The waiting room setting may have affected response quality and completeness due to varying time constraints, including frequent usage of “not specified” as an answer. The short lifespan of internet content and design complicate long‐term validity of assessments. The single‐prompt approach in LLM evaluation does not reflect typical multi‐turn interactions with LLMs, potentially underestimating their capabilities. A single calculator for readability was used, which might over‐ or underestimate the real readability, because several calculators may provide different results [25].

## 6 Conclusions

Online resources have become a cornerstone of patient information in andrology, despite variable quality. This study highlights the need for improvement in official medical association websites, particularly in readability and comprehensibility, to provide reliable, high‐quality information that physicians can confidently recommend.

Healthcare providers should recognize patients’ online research as an opportunity for enhanced patient education and empowerment. However, the physician–patient relationship remains fundamental to medical communication. Our findings underscore the critical need for improved education in online media literacy and communication skills for healthcare professionals.

## Author Contributions

MT: research design, data acquisition, data analysis, data interpretation, paper draft, paper revision. PG: data acquisition, data analysis, data interpretation, paper draft. JH, JK and SM: data acquisition, paper revision. RH: data analysis, paper revision. CG: research design, paper revision. DS: research design, data interpretation, paper draft, paper revision.

## Funding

Traeger, Max: Funded by the Berta‐Ottenstein‐Programme for Clinician Scientists, Faculty of Medicine, University of Freiburg.

## Disclosure

All authors declare no financial or non‐financial interests that are directly or indirectly related to the work submitted for publication.

## Consent

No written consent has been obtained from the patients as there is no patient identifiable data included.

## Supporting information




**Supporting File 1**: andr70286‐sup‐0001‐SuppMat.docx.
