# Supplementary material for: Online Andrology: Real‐World Status Quo of Patients’ Online Information Sources and Comparison With Large Language Models in Germany
Source: Andrology. 2026 Jun 22;14(6):1823–30. doi: 10.1111/andr.70286 (PMC13432683; doi:10.1111/andr.70286)
Supplement: Supplementary file 1 — Supporting File 1: andr70286‐sup‐0001‐SuppMat.docx. [file ANDR-14-1823-s001.docx]

**Supplementary Material**

S1: German questionnaire

See separate file

S2: Queries for LLMs

Erektile Dysfunction:

1. Woher kommen Erektionsprobleme?
2. Welche Therapie gibt es gegen Erektionsprobleme?
3. Werden die Erektionen wieder besser?

Induratio penis plastica:

1. Warum ist mein Penis krumm?
2. Welche Therapie gibt es gegen eine Penisverkrümmung?
3. Wird ein krummer Penis von alleine wieder gerade?

Fertility:

1. Wie kann ich herausfinden, warum ich als Mann keine Kinder zeugen kann?
2. Welche Therapie gibt es, wenn man als Mann keine Kinder zeugen kann?
3. Gibt es eine Chance mit schlechten Spermien Kinder zu zeugen?

S3: Participants’ educational background

S4: Most common internet sources

S5: DISCERN analysis for erectile dysfunction

| **Website** | **Link** | **Date** | **DISCERN Score** |
| --- | --- | --- | --- |
| Apotheken Umschau | https://www.apotheken-umschau.de/krankheiten-symptome/erkrankungen-der-maennlichen-geschlechtsorgane/erektile-dysfunktion-ursachen-und-behandlung-737345.html | 04.06.2023; 17:05 | 69 |
| Netdoktor | https://www.netdoktor.de/symptome/impotenz/ | 11.11.2023; 14:49 | 64 |
| Wikipedia | <https://de.wikipedia.org/wiki/Erektile_Dysfunktion> | 04.06.2023; 17:42 | 60 |
| MSD Manual | https://www.msdmanuals.com/de-de/heim/gesundheitsprobleme-von-m%C3%A4nnern/sexuelle-funktionsst%C3%B6rungen-bei-m%C3%A4nnern/erektionsst%C3%B6rung-erektile-dysfunktion-ed?query=erektile%20dysfunktion | 04.06.2023; 18:49 | 59 |
| Doccheck | https://flexikon.doccheck.com/de/Erektile_Dysfunktion | 11.11.2023; 17:16 | 38 |
| DGU | <https://urologische-stiftung-gesundheit.de/ratgeber/erektionsstoerungen/> | 04.06.2023; 19:25 | 37 |

S6: DISCERN analysis for penile deviation

| **Website** | **Link** | **Date** | **DISCERN Score** |
| --- | --- | --- | --- |
| Wikipedia | <https://de.wikipedia.org/wiki/Induratio_penis_plastica> | 10.11.2023; 17:39 Uhr | 71 |
| Doccheck | https://flexikon.doccheck.com/de/Induratio_penis_plastica | 11.11.2023; 12:27 Uhr | 71 |
| Netdoktor | https://www.netdoktor.de/krankheiten/penisverkruemmung/ | 11.11.2023; 14:05 Uhr | 70 |
| MSD Manual | <https://www.msdmanuals.com/de-de/heim/gesundheitsprobleme-von-m%C3%A4nnern/penis-und-hodenerkrankungen/peyronie-krankheit?query=induratio%20penis%20plastica> | 10.11.2023; 19:51 Uhr | 53 |
| Apotheken Umschau | <https://www.apotheken-umschau.de/krankheiten-symptome/symptome/schmerzen-beim-sex-ursachen-bei-maennern-741431.html#penisverhrtung-induratio-penis-plastica-ipp> | 10.11.2023; 19:12 Uhr | 41 |
| DGU | https://urologische-stiftung-gesundheit.de/organe-und-haeufige-erkrankungen/haeufige-erkrankungen-des-penis/ | 16.11.2023; 12:10 Uhr | 22 |

S7: DISCERN analysis for fertility problems

| **Website** | **Link** | **Date** | **DISCERN Score** |
| --- | --- | --- | --- |
| Netdoktor | https://www.netdoktor.de/kinderwunsch/unfruchtbarkeit/des-mannes/ | 16.11.2023; 15:16 | 57 |
| Wikipedia | <https://de.wikipedia.org/wiki/Unfruchtbarkeit> | 19.11.2023; 13:35 | 57 |
| MSD Manual | https://www.msdmanuals.com/de-de/heim/gesundheitsprobleme-von-frauen/unfruchtbarkeit/probleme-mit-den-spermien | 16.11.2023; 14:56 | 56 |
| Doccheck | https://flexikon.doccheck.com/de/Unfruchtbarkeit | 19.11.2023; 15:14 | 39 |
| Apotheken Umschau | <https://www.apotheken-umschau.de/familie/kinderwunsch/unerfuellter-kinderwunsch/ungewollt-kinderlos-nicht-selten-liegt-s-am-unfruchtbaren-mann-885645.html> | 19.11.2023; 14:40 | 38 |
| DGU | https://urologische-stiftung-gesundheit.de/erkrankungen/andrologische-erkrankungen | 16.11.2023; 12:16 | 31 |

S8: Flesch analysis for erectile dysfunction

| **Website** | **Link** | **Date** | **Flesch-Index** |
| --- | --- | --- | --- |
| Netdoktor | https://www.netdoktor.de/symptome/impotenz/ | 11.11.2023; 14:49 | 40 |
| Apotheken Umschau | https://www.apotheken-umschau.de/krankheiten-symptome/erkrankungen-der-maennlichen-geschlechtsorgane/erektile-dysfunktion-ursachen-und-behandlung-737345.html | 04.06.2023; 17:05 | 37 |
| Wikipedia | <https://de.wikipedia.org/wiki/Erektile_Dysfunktion> | 04.06.2023; 17:42 | 32 |
| MSD Manual | https://www.msdmanuals.com/de-de/heim/gesundheitsprobleme-von-m%C3%A4nnern/sexuelle-funktionsst%C3%B6rungen-bei-m%C3%A4nnern/erektionsst%C3%B6rung-erektile-dysfunktion-ed?query=erektile%20dysfunktion | 04.06.2023; 18:49 | 32 |
| DGU | <https://urologische-stiftung-gesundheit.de/ratgeber/erektionsstoerungen/> | 04.06.2023; 19:25 | 30 |
| Doccheck | https://flexikon.doccheck.com/de/Erektile_Dysfunktion | 11.11.2023; 17:16 | 12 |

S9: Flesch analysis for penile deviation

| **Website** | **Link** | **Date** | **Flesch-Index** |
| --- | --- | --- | --- |
| Apotheken Umschau | <https://www.apotheken-umschau.de/krankheiten-symptome/symptome/schmerzen-beim-sex-ursachen-bei-maennern-741431.html#penisverhrtung-induratio-penis-plastica-ipp> | 10.11.2023; 19:12 Uhr | 42 |
| Netdoktor | https://www.netdoktor.de/krankheiten/penisverkruemmung/ | 11.11.2023; 14:05 Uhr | 37 |
| MSD Manual | <https://www.msdmanuals.com/de-de/heim/gesundheitsprobleme-von-m%C3%A4nnern/penis-und-hodenerkrankungen/peyronie-krankheit?query=induratio%20penis%20plastica> | 10.11.2023; 19:51 Uhr | 36 |
| DGU | https://urologische-stiftung-gesundheit.de/organe-und-haeufige-erkrankungen/haeufige-erkrankungen-des-penis/ | 16.11.2023; 12:10 Uhr | 32 |
| Wikipedia | <https://de.wikipedia.org/wiki/Induratio_penis_plastica> | 10.11.2023; 17:39 Uhr | 31 |
| Doccheck | https://flexikon.doccheck.com/de/Induratio_penis_plastica | 11.11.2023; 12:27 Uhr | 19 |

S10: Flesch analysis for fertility problems

| **Website** | **Link** | **Date** | **Flesch-Index** |
| --- | --- | --- | --- |
| Apotheken Umschau | <https://www.apotheken-umschau.de/familie/kinderwunsch/unerfuellter-kinderwunsch/ungewollt-kinderlos-nicht-selten-liegt-s-am-unfruchtbaren-mann-885645.html> | 19.11.2023; 14:40 | 56 |
| MSD Manual | <https://www.msdmanuals.com/de-de/heim/gesundheitsprobleme-von-frauen/unfruchtbarkeit/probleme-mit-den-spermien> | 16.11.2023; 14:56 | 34 |
| Netdoktor | https://www.netdoktor.de/kinderwunsch/unfruchtbarkeit/des-mannes/ | 16.11.2023; 15:16 | 32 |
| Wikipedia | <https://de.wikipedia.org/wiki/Unfruchtbarkeit> | 19.11.2023; 13:35 | 25 |
| DGU | https://urologische-stiftung-gesundheit.de/erkrankungen/andrologische-erkrankungen/ | 16.11.2023; 12:16 | 22 |
| Doccheck | https://flexikon.doccheck.com/de/Unfruchtbarkeit | 19.11.2023; 15:14 | 15 |
